# New Appliances and Things Medical

**Published:** 1907-03-09

**Authors:** 


					new appliances and things medical.
EIFFEL TOWER. JU-VIS (ORIGINALLY KNOW X
AS OX-CUP).
(G. Foster Clarke and Co., Maipsxoke.) _
This remarkably cheap preparation is so ^^
varying in price from*Id. to lO^d. u!?latine_iike tablets,
?each other by containing one 01 m g, ^ make from
a single tablet costing one pennj. gQ t^ie
the preparation the corresponding beef- shouid be
tablets should be cut up into small pieces a
dissolved in about six or eight ounces o boihng
The product has a very similar taste anc p y other
lately the same dietetic properties as the
varieties of beef-tea so called 011 the market. these
however, to contain more gelatine than is _ kitchen
Preparations, and is consequently of value u ^ It
for increasing the setting capacity of co c ?ta ' , can
forms also an excellent basis for ordinary sou ^
be made by suitable means either into a uc tvpe
"the addition of chopped vegetable into a sour> we must
?? julienne. From the point of its nutritive > though
regard it more as a stimulant than as a 1111 ^ limited
the gelatine which it contains may be regarc e 11
?sense as an actual food.

				

